# Supplementary material for: Discovery and application of insertion-deletion (INDEL) polymorphisms for QTL mapping of early life-history traits in Atlantic salmon
Source: BMC Genomics. 2010 Mar 8;11:156. doi: 10.1186/1471-2164-11-156 (PMC2838853; doi:10.1186/1471-2164-11-156)
Supplement: Additional file 2 — Information on developed 76 locus single-run INDEL panel in Atlantic salmon. Information on fluorescence labeling, primer concentrations, PCR pooling and links to alignments, INDEL motifs and GENESCAN (Burge and Karlin 1997) predictions of genes/exons are available in html format. [file 1471-2164-11-156-S2.ZIP › Additionalfile2/table18185.html]

Contig Alignment

```
Base	A B C D E F   
1	. G . . . .    
2	. A . . . .    
3	. G . . . .    
4	. T . . . .    
5	. T . . . .    
6	. A . . . .    
7	. C . C . .    
8	. A . A . .    
9	. G . G . .    
10	. T . T . .    
11	. C . C . .    
12	. A . A . .    
13	. C . C . .    
14	. C . C . .    
15	. C . C . .    
16	. T . T . .    
17	. G . G . .    
18	. C . C . .    
19	. T . T . .    
20	. G . G . .    
21	. T . T . .    
22	. C . C . .    
23	. T . T . .    
24	. T . T . .    
25	. A . A . .    
26	. A . A . .    
27	. C . C . .    
28	. G . G . .    
29	. C . C . .    
30	. G . G . .    
31	. G . G . .    
32	. G . G . .    
33	. A . A . .    
34	. C . C . .    
35	. A . A . .    
36	. C . C . .    
37	. A . A . .    
38	. T . T . .    
39	. C . C . .    
40	. C . C . .    
41	. C . C . .    
42	. G . G . .    
43	. C . C . .    
44	. A . A . .    
45	. T . T . .    
46	. T . T . .    
47	. C . C . .    
48	. C . C . .    
49	. T . T . .    
50	. C . C . .    
51	. T . T . .    
52	. C . C . .    
53	. C . C . .    
54	. T . T . .    
55	. C . C . .    
56	. A . A . .    
57	. A . A . .    
58	. C . C . .    
59	. C . C . .    
60	. A . A . .    
61	. G . G . .    
62	. A . A . .    
63	. A . A . .    
64	. G . G . .    
65	. G . G . .    
66	. C . C . .    
67	. T . T . .    
68	. G . G . .    
69	. G . G . .    
70	. C . C . .    
71	. T . T . .    
72	. T . T . .    
73	. T . T . .    
74	. C . C . .    
75	. C . C . .    
76	. A . A . .    
77	. G . G . .    
78	. A . A . .    
79	. G . G . .    
80	. A . A . .    
81	. A . A . .    
82	. A . A . .    
83	. G . G . .    
84	. A . A . .    
85	. T . T . .    
86	. G . G . .    
87	. T . T . .    
88	. T . T . .    
89	. G . G . .    
90	. C . C . .    
91	. C . C . .    
92	. T . T . .    
93	. C . C . .    
94	. A . A . .    
95	. C . C . .    
96	. T . T . .    
97	. G . G . .    
98	. A . A A .    
99	. A . A A .    
100	. A . A A .    
101	. A . A A .    
102	. G . G G .    
103	. A . A A .    
104	. C . C C .    
105	. T . T T .    
106	. C . C C .    
107	. A . A A .    
108	. C . C C .    
109	. C . C C .    
110	. A . A A .    
111	. A . A A .    
112	. G . G G .    
113	. G . G G .    
114	. C . C C .    
115	. C . C C .    
116	. C . C C .    
117	. T . T T .    
118	. G . G G .    
119	. T . T T .    
120	. T . T T .    
121	. C . C C .    
122	. A . A A .    
123	. A . A A .    
124	. C . C C .    
125	. C . C C C    
126	. G . G G G    
127	. C . C C C    
128	. A . A A A    
129	. A . A A A    
130	. C . C C C    
131	. A . A A A    
132	. T . T T T    
133	. C . C C C    
134	. C . C C C    
135	. A . A A A    
136	. G . G G G    
137	. A . A A A    
138	. C . C C C    
139	. T . T T T    
140	. G . G G G    
141	. G . G G G    
142	. G . G G G    
143	. A . A A A    
144	. A . A A A    
145	. G . G G G    
146	. A . A A A    
147	. A . A A A    
148	. G . G G G    
149	. G . G G G    
150	. G . G G G    
151	. G . G G G    
152	. C . C C C    
153	. A . A A A    
154	. C . C C C    
155	. T . T T T    
156	. C . C C C    
157	. G . G G G    
158	. T . T T T    
159	. C . C C C    
160	. C . C C C    
161	. G . G G G    
162	. T . T T T    
163	. G . G G G    
164	. G . G G G    
165	. A . A A A    
166	. G . G G G    
167	. G . G G G    
168	. A . A A A    
169	. T . T T T    
170	. G . G G G    
171	. C . C C C    
172	. C . C C C    
173	. A . A A A    
174	. A . A A A    
175	. A . A A A    
176	. G . G G G    
177	. C . C C C    
178	. C C C C C    
179	. A A A A A    
180	. C C C C C    
181	. C C C C C    
182	. A A A A A    
183	. T T T T T    
184	. G G G G G    
185	. G G G G G    
186	. A A A A A    
187	. A A A A A    
188	. C C C C C    
189	. T T T T T    
190	. G G G G G    
191	. T T T T T    
192	. A A A A A    
193	. C C C C C    
194	. A A A A A    
195	. A A A A A    
196	. A A A A A    
197	. G G G G G    
198	. T T T T T    
199	. T T T T T    
200	. G G G G G    
201	. T T T T T    
202	. G G G G G    
203	. G G G G G    
204	. A A A A A    
205	. G G G G G    
206	. G G G G G    
207	. T T T T T    
208	. G G G G G    
209	. G G G G G    
210	. A A A A A    
211	. G G G G G    
212	. T T T T T    
213	. G G G G G    
214	. G G G G G    
215	. G G G G G    
216	. A A A A A    
217	. G G G G G    
218	. A A A A A    
219	. G G G G G    
220	. G G G G G    
221	. A A A A A    
222	. C C C C C    
223	. C C C C C    
224	. T T T T T    
225	. T T T T T    
226	. A A A A A    
227	. G G G G G    
228	. C C C C C    
229	. C C C C C    
230	. T T T T T    
231	. C C C C C    
232	. C C C C C    
233	. A A A A A    
234	. A A A A A    
235	. A A A A A    
236	. T T T T T    
237	. A A A A A    
238	. A A A A A    
239	. A A A A A    
240	. A A A A A    
241	. A A A A A    
242	. A A A A A    
243	. A A A A A    
244	. G G G G G    
245	. C C C C C    
246	. C C C C C    
247	. A A A A A    
248	. G G G G G    
249	. T T T T T    
250	. G G G G G    
251	. A A A A A    
252	. A A A A A    
253	. A A A A A    
254	. G G G G G    
255	. C C C C C    
256	. C C C C C    
257	. T T T T T    
258	. G G G G G    
259	. T T T T T    
260	. A A A A A    
261	. T T T T T    
262	. G G G G G    
263	. G G G G G    
264	. A A A A A    
265	. C C C C C    
266	T T T T T T    
267	G G G G G G    
268	A A A A A A    
269	A A A A A A    
270	C C C C C C    
271	T T T T T T    
272	G G G G G G    
273	G G G G G G    
274	T T T T T T    
275	G G G G G G    
276	A A A A A A    
277	A A A A A A    
278	A A A A A A    
279	G G G G G G    
280	G G G G G G    
281	G G G G G G    
282	T T T T T T    
283	T T T T T T    
284	G G G G G G    
285	A A A A A A    
286	G G G G G G    
287	A A A A A A    
288	G G G G G G    
289	A A A A A A    
290	T T T T T T    
291	G G G G G G    
292	G G G G G G    
293	T T T T T T    
294	A A A A A A    
295	T T T T T T    
296	G G G G G G    
297	G G G G G G    
298	A A A A A A    
299	C C C C C C    
300	G G G G G G    
301	T T T T T T    
302	A A A A A A    
303	T T T T T T    
304	C C C C C C    
305	A A A A A A    
306	T T T T T T    
307	G G G G G G    
308	T T T T T T    
309	T T T T T T    
310	T T T T T T    
311	A A A A A A    
312	A A A A A A    
313	C C C C C C    
314	A A A A A A    
315	T T T T T T    
316	A A A A A A    
317	A A A A A A    
318	T T T T T T    
319	T T T T T T    
320	T T T T T T    
321	G G G G G G    
322	A A A A A A    
323	A A A A A A    
324	C C C C C C    
325	A A A A A A    
326	T T T T T T    
327	T T T T T T    
328	C C C C C C    
329	A A A A A A    
330	G G G G G G    
331	A A A A A A    
332	A A A A A A    
333	A A A A A A    
334	T T T T T T    
335	C C C C C C    
336	A A A A A A    
337	C C C C C C    
338	T T T T T T    
339	A A A A A A    
340	T T T T T T    
341	C C C C C C    
342	A A A A A A    
343	G G G G G G    
344	G G G G G G    
345	T T T T T T    
346	C C C C C C    
347	T T T T T T    
348	T T T T T T    
349	C C C C C C    
350	C C C C C C    
351	A A A A A A    
352	C C C C C C    
353	T T T T T T    
354	T T T T T T    
355	C C C C C C    
356	T T T T T T    
357	C C C C C C    
358	A A A A A A    
359	C C C C C C    
360	C C C C C C    
361	T T T T T T    
362	C C C C C C    
363	A A A A A A    
364	G G G G G G    
365	C C C C C C    
366	C C C C C C    
367	A A A A A A    
368	C C C C C C    
369	T T T T T T    
370	T T T T T T    
371	C C C C C C    
372	T T T T T T    
373	G G G G G G    
374	T T T T T T    
375	C C C C C C    
376	T T T T T T    
377	C C C C C C    
378	T T T T T T    
379	T T T T T T    
380	T T T T T T    
381	T T T T T T    
382	T T T T T T    
383	C C C C C C    
384	T T T T T T    
385	A A A A A A    
386	C C C C C C    
387	T T T T T T    
388	T T T T T T    
389	C C C C C C    
390	C C C C C C    
391	G G G G G G    
392	T T T T T T    
393	A A A A A A    
394	A A A A A A    
395	A A A A A A    
396	A A A A A A    
397	A A A A A A    
398	A - - - - A  2	 indel  
399	A - - - - A  2	 indel  
400	A - - - - A  2	 indel  
401	C C C C C C    
402	A A A A A A    
403	G G G G G G    
404	T T T T T T    
405	T T T T T T    
406	G G G G G G    
407	C C C C C C    
408	C C C C C C    
409	T T T T T T    
410	T T T T T T    
411	C C C C C C    
412	A A A A A A    
413	G G G G G G    
414	G G G G G G    
415	G G G G G G    
416	T T T T T T    
417	T T T T T T    
418	G G G G G G    
419	C C C C C C    
420	A A A A A A    
421	G G G G G G    
422	T T T T T T    
423	T T T T T T    
424	G G G G G G    
425	T T T T T T    
426	T T T T T T    
427	T T T T T T    
428	A A A A A A    
429	T T T T T T    
430	T T T T T T    
431	T T T T T T    
432	T T T T T T    
433	G G G G G G    
434	T T T T T T    
435	G G G G G G    
436	G G G G G G    
437	T T T T T T    
438	G G G G G G    
439	G G G G G G    
440	A A A A A A    
441	T T T T T T    
442	G G G G G G    
443	A A A A A A    
444	C C C C C C    
445	C C C C C C    
446	T T T T T T    
447	A A A A A A    
448	T T T T T T    
449	G G G G G G    
450	G G G G G G    
451	C C C C C C    
452	A A A A A A    
453	C C C C C C    
454	A A A A A A    
455	A A A A A A    
456	G G G G G G    
457	A A A A A A    
458	T T T T T T    
459	G G G G G G    
460	G G G G G G    
461	C C C C C C    
462	A A A A A A    
463	G G G G G G    
464	T T T T T T    
465	C C C C C C    
466	C C C C C C    
467	T T T T T T    
468	C C C C C C    
469	T T T T T T    
470	A A A A A A    
471	C C C C C C    
472	C C C C C C    
473	A A A A A A    
474	C C C C C C    
475	A A A A A A    
476	A A A A A A    
477	G G G G G G    
478	G G G G G G    
479	G G G G G G    
480	T T T T T T    
481	G G G G G G    
482	C C C C C C    
483	A A A A A A    
484	G G G G G G    
485	T T T T T T    
486	C C C C C C    
487	T T T T T T    
488	A A A A A A    
489	T T T T T T    
490	T T T T T T    
491	T T T T T T    
492	T T T T T T    
493	C C C C C C    
494	T T T T T T    
495	C C C C C C    
496	G G G G G G    
497	G G G G G G    
498	T T T T T T    
499	T T T T T T    
500	T T T T T T    
501	T T T T T T    
502	A A A A A A    
503	T T T T T T    
504	T T T T T T    
505	A A A A A A    
506	A A A A A A    
507	T T T T T T    
508	G G G G G G    
509	A A A A A A    
510	A A A A A A    
511	T T T T T T    
512	T T T T T T    
513	C C C C C C    
514	C C C C C C    
515	T T T T T T    
516	A A A A A A    
517	A A A A A A    
518	T T T T T T    
519	A A A A A A    
520	C C C C C C    
521	A A A A A A    
522	G G G G G G    
523	T T T T T T    
524	A A A A A A    
525	C C C C C C    
526	T T T T T T    
527	G G G G G T    
528	T T T T T T    
529	C C C C C C    
530	A A A A A A    
531	T T T T T T    
532	G G G G G G    
533	T T T T T T    
534	T T T T T T    
535	G G G G G G    
536	T T T T T T    
537	T T T T T T    
538	T T T T T T    
539	T T T T T T    
540	G G G G G G    
541	C C C C C C    
542	T T T T T T    
543	T T T T T T    
544	T T T T T T    
545	G G G G G G    
546	A A A A A A    
547	A A A A A A    
548	T T T T T T    
549	T T T T T T    
550	G G G G G G    
551	T T T T T T    
552	C C C C C C    
553	T T T T T T    
554	A A A A A A    
555	G G G G G G    
556	A A A A A A    
557	G G G G G G    
558	T T T T T T    
559	T T T T T T    
560	A A A A A A    
561	T T T T T T    
562	T T T T T T    
563	T T T T T T    
564	G G G G G G    
565	T T T T T T    
566	T T T T T T    
567	C C C C C C    
568	A A A A A A    
569	G G G G G G    
570	G G G G G G    
571	C C C C C C    
572	A A A A A A    
573	T T T T T T    
574	T T T T T T    
575	T T T T T T    
576	C C C C C C    
577	T T T T T T    
578	A A A A A A    
579	T T T T T T    
580	T T T T T T    
581	T T T T T T    
582	T T T T T T    
583	A A A A A A    
584	T T T T T T    
585	A A A A A A    
586	T T T T T T    
587	A A A A A A    
588	T T T T T T    
589	T T T T T T    
590	A A A A A A    
591	A A A A A A    
592	A A A A A A    
593	T T T T T T    
594	T T T T T T    
595	G G G G G G    
596	T T T T T T    
597	T T T T T T    
598	A A A A A A    
599	A A A A A A    
600	C C C C C C    
601	A A A A A A    
602	T T T T T T    
603	A A A A A A    
604	C C C C C C    
605	A A A A A A    
606	G G G G G G    
607	T T T T T T    
608	A A A A A A    
609	T T T T T T    
610	C C C C C C    
611	T T T T T T    
612	C C C C C C    
613	A A A A A A    
614	G G G G G G    
615	T T T T T T    
616	A A A A A A    
617	C C C C C C    
618	A A A A A A    
619	T T T T T T    
620	C C C C C C    
621	A A A A A A    
622	. T T T T T    
623	. G G G G G    
624	. T T T T T    
625	. A A A A A    
626	. C C C C C    
627	. A A A A A    
628	. T T T T T    
629	. G G G G G    
630	. T T T T T    
631	. T T T T T    
632	. C C C C C    
633	. A A A A A    
634	. T T T T T    
635	. T T T T T    
636	. T T T T T    
637	. T T T T T    
638	. G G G G G    
639	. A A A A A    
640	. C C C C C    
641	. A A A A A    
642	. G G G G G    
643	. G G G G G    
644	. T T T T T    
645	. G G G G G    
646	. A A A A A    
647	. A A A A A    
648	. A A A A A    
649	. G G G G G    
650	. T T T T T    
651	. A A A A A    
652	. A A A A A    
653	. A A A A A    
654	. A A A A A    
655	. T T T T T    
656	. A A A A A    
657	. G G G G G    
658	. A A A A A    
659	. T T T T T    
660	. T T T T T    
661	. T T T T T    
662	. T T T T T    
663	. A A A A A    
664	. A A A A A    
665	. T T T T T    
666	. A A A A A    
667	. C C C C C    
668	. A A A A A    
669	. A A A A A    
670	. C C C C C    
671	. T T T T T    
672	. G G G G G    
673	. C C C C C    
674	. A A A A A    
675	. G G G G G    
676	. T T T T T    
677	. A A A A A    
678	. T T T T T    
679	. A A A A A    
680	. A A A A A    
681	. T T T T T    
682	. G G G G G    
683	. A A A A A    
684	. A A A A A    
685	. G G G G G    
686	. A A A A A    
687	. A A A A A    
688	. A A A A A    
689	. T T T T T    
690	. T T T T T    
691	. G G G G G    
692	. G G G G G    
693	. T T T T T    
694	. T T T T T    
695	. T T T T T    
696	. T T T T T    
697	. A A A A A    
698	. A A A A A    
699	. A A A A A    
700	. C C C C C    
701	. A A A A A    
702	. A A A A A    
703	. T T T T T    
704	. T T T T T    
705	. A A A A A    
706	. T T T T T    
707	. T T T T T    
708	. G G G G G    
709	. T T T T T    
710	. . A A A A    
711	. . A A A A    
712	. . T T T T    
713	. . G G G G    
714	. . G G G G    
715	. . C C C C    
716	. . A A A A    
717	. . A A A A    
718	. . A A A A    
719	. . C C C C    
720	. . A A A A    
721	. . G T T T    
722	. . . G G G    
723	. . . G G G    
724	. . . C C C    
725	. . . A A A    
726	. . . A A .    
727	. . . . A .    
728	. . . . A .    
729	. . . . A .    
730	. . . . G .    
731	. . . . A .    
732	. . . . A .    
733	. . . . A .    
734	. . . . T .    
735	. . . . A .    
736	. . . . A .    
737	. . . . A .    
738	. . . . T .    
739	. . . . G .    
740	. . . . A .    
741	. . . . C .    
742	. . . . C .    
743	. . . . A .    
744	. . . . T .    
745	. . . . T .    
746	. . . . G .    
747	. . . . C .    
748	. . . . A .    
749	. . . . A .    
750	. . . . A .    
751	. . . . C .    
752	. . . . T .    
753	. . . . T .    
754	. . . . T .    
755	. . . . T .
```
